# Endothelial and mural laminin-α5 contributes to neurovascular integrity maintenance

**DOI:** 10.1186/s12987-024-00521-z

**Published:** 2024-02-21

**Authors:** Abhijit Nirwane, Minkyung Kang, Aravinthan Adithan, Vrishni Maharaj, Felicia Nguyen, Elliot Santaella Aguilar, Ava Nasrollahi, Yao Yao

**Affiliations:** Department of Molecular Pharmacology and Physiology, Morsani College of Medicine, University of South Florida, 12901 Bruce B. Downs Blvd., MDC 8, 33612 Tampa, FL USA

**Keywords:** Neurovascular unit, Transcytosis, Endothelial cells, Mural cells, Laminin-α5

## Abstract

**Background:**

Laminin-α5, a major component of the basal lamina, is predominantly synthesized by endothelial and mural cells (pericytes and vascular smooth muscle cells) in the CNS. Loss of laminin-α5 in either population fails to induce any abnormalities due to functional redundancy. Thus, the functional significance of laminin-α5 in neurovascular integrity remains unknown. Here, we hypothesize that ablation of laminin-α5 in both endothelial and mural cells increases neurovascular permeability.

**Methods:**

The compound knockout mice were generated by crossing laminin-α5 floxed mice with Tie2-Cre and PDGFRβ-Cre, which target endothelial cells and mural cells, respectively. Neurovascular permeability in these mutants was determined with both exogenous and endogenous tracers. Endothelial paracellular and transcellular permeability was assessed by examining the expression of tight junction proteins and transcytosis-associated proteins. In addition, transmission electron microscopy (TEM) was used to visualize tight junction ultrastructure and endothelial caveolae vesicles. Defects in pericytes and astrocytes were investigated by examining pericyte coverage/contact and astrocyte polarity.

**Results:**

Elevated neurovascular permeability was observed in the mutants. Subsequent studies found increased Caveolin-1 and decreased major facilitator superfamily domain-containing protein 2a (MFSD2A) expression, but unaltered Claudin-5 or zonula occludens-1 (ZO-1) expression. Consistent with these results, mutant mice exhibited increased endothelial caveolae vesicle number with intact tight junction structure under TEM. Additionally, pericyte coverage and contact were also decreased in the mutant mice, while astrocyte polarity was unaffected.

**Conclusions:**

These results strongly indicate that endothelial and mural cell-derived laminin-α5 actively maintains neurovascular integrity via the transcellular rather than paracellular mechanism.

## Background

Neurovascular unit (NVU) is a dynamic structure located at the interface of the CNS and circulation system [1, 2]. By tightly regulating what enters and leaves the brain, it plays an important role in maintaining brain homeostasis. Not only is NVU disfunction observed in most neurological disorders, it also actively contributes to the pathogenesis of many of them [2, 3]. NVU is mainly composed of endothelial cells, pericytes, astrocyte endfeet, and a non-cellular component—the basal lamina [1, 2]. Although how different cells regulate neurovascular integrity is well studied, the role of the basal lamina in neurovascular integrity remains largely unknown.

Laminin, the only component required for basal lamina assembly, is a heterotrimeric protein containing α, β, and γ subunits [4–6]. Among the five α subunits, laminin-α2, -α4, and -α5 are expressed in vascular cells. Specifically, astrocytes make laminin-α2β1γ1 (-211) [4, 5, 7, 8]; endothelial cells mainly synthesize laminin-411 and − 511 [4, 5, 7, 9]; and mural cells predominantly generate laminin-211, -411, -511, and possibly 221, 421, 521 [4, 5, 10, 11]. Loss-of-function studies in mice have found an indispensable and negligible role of laminin-α2 [12–14] and laminin-α4 [15] in neurovascular integrity maintenance, respectively. Deletion of laminin-α5 globally results in multi-organ defects and embryonic lethality in mice [16], preventing the investigation of neurovascular integrity at later stages. In humans, heterogenous *Lama5* mutations have been associated with infant epilepsy, skin anomalies, impaired scarring, night blindness, muscle weakness, osteoarthritis, joint and internal organs ligaments laxity, malabsorption syndrome and hypothyroidism [17, 18]. Homozygous *Lama5* mutations have been linked to bent bone dysplasia [19], neuromuscular transmission defects [20], and a complex syndromic disease characterized by kidney defects and abnormal craniofacial & limb development [21]. To investigate laminin-α5’s function in neurovascular integrity, we have generated various laminin-α5 conditional knockout mouse lines. Interestingly, endothelial [22] or mural [23] cell-specific laminin-α5 knockout mice fail to show increased neurovascular permeability or other abnormalities under homeostatic conditions, highlighting a dispensable role of endothelial or mural cell-derived laminin-α5.

Considering potential functional redundancy, we hypothesize that endothelial and mural cell-derived laminin-α5 may be able to compensate for each other’s loss. To test this hypothesis and investigate the functional significance of laminin-α5 in neurovascular integrity, we further generated compound mutant mice with laminin-α5 deficiency in both endothelial and mural cells and characterized their neurovascular integrity in this study.

## Methods

### Mice

The Laminin-α5^flox/flox^ mice [24] and platelet-derived growth factor receptor beta (PDGFRβ)-Cre^+^ mice [25] were generous gifts from Drs. Jeffrey Miner and Volkhard Lindner, respectively. The Tie2-Cre^+^ (Jax:008863) mice were obtained from the Jackson Laboratory. The α5-TPKO (Laminin-α5^flox/flox^;PDGFRβ-Cre^+^;Tie2-Cre^+^) mice were generated by crossing the above three mouse lines. Since PDGFRβ-Cre^+^;Tie2-Cre^+^ mice failed to show obvious defects and are indistinguishable from Laminin-α5^flox/flox^ mice, both were used as controls for α5-TP-KO mice. All mice were maintained on a C57Bl6 background. Mice (both sexes) at the age of 4∼6 months were used in this study. Mice were housed in the animal facility of the University of South Florida and provided with ad libitum access to food and water. The experimental protocols and procedures of this study were approved by the Institutional Animal Care and Use Committee at the University of South Florida and were conducted in accordance with the National Institutes of Health’s Guide for the Care and Use of Laboratory Animals.

### Genotyping

Collected mouse samples were subjected to genomic DNA isolation, followed by PCR amplification. To differentiate Tie2-Cre and PDGFRβ-Cre, promoter-specific primers were used. The primers used in this study are shown below. Tie2-Cre-Forward: GACTGTTACCGCCTGCTTCT; Tie2-Cre-Reverse: GTTCTGCGGGAAACCATTT; PDGFRβ-Cre-Forward: GGATGCTTTTGGAGTGAGGAG; PDGFRβ-Cre-Reverse: CCAGGTATGCTCAGAAAACGC; Laminin-α5-Forward: CTGCCGCCCTAACACCCAAGG; Laminin-α5-Reverse: GTTGAAGCCAAAGCGTACAGCG.

### In vivo permeability assay

Neurovascular permeability was assessed in vivo as described in our previous publications [10, 12, 26, 27]. For exogenous marker, 50ul of 5 mg/ml Sulfo-NHS-Biotin (ThermoFisher, 21,217) in sterile saline was injected into control and α5-TPKO mice intravenously. After 6 h of circulation, mice were transcardially perfused with 4% PFA. In brain sections, Sulfo-NHS-Biotin was visualized with Avidin-FITC (1:200, BD, 554,057). For endogenous marker, Hemoglobin was detected by immunohistochemistry. Sulfo-NHS-Biotin and Hemoglobin were co-stained with vascular marker Podocalyxin. For quantifications, three random fields from each section, 8 serial sections along the rostral-to-caudal axis of each brain, and 5 animals were used. Due to the small number of leakage spots in each brain region, tracer leakage was determined using the whole brain to enable a more accurate quantification.

### Tissue processing

Mice were anesthetized by intraperitoneal injection of inactin (100 mg/kg body weight), followed by transcardiac perfusion with PBS and/or 4% PFA. Brains were collected and subjected to serial sectioning. Briefly, 20 μm-thick serial sections were cut with Cryostat (Micro HM 550, Thermo Scientific), and eight sections evenly distributed along the rostral-to-caudal axis were collected from each brain. Brain sections were stored at − 80 °C until use.

### Immunohistochemistry and immunocytochemistry

Immunohistochemistry was performed using a standard protocol. Briefly, brain sections were fixed in 4% PFA for 15 min at room temperature. After extensive washes, brain sections were blocked in blocking buffer (5% normal donkey serum, 3% BSA, 0.3% Triton X-100 in PBS) for 1 h and incubated with primary antibodies overnight at 4 °C. The following primary antibodies were used: anti-Laminin-α1 (1:200, R&D, MAB4656), anti-Laminin-α2 (1:300, Sigma, L0663), anti-Laminin-α4 (1:300, R&D, AF3837), anti-Laminin α5 (1:800, a generous gift from Dr. Jeffrey Miner), anti-Nidogen (1:200, Invitrogen, MA1-06501), anti-Collagen IV (1:400, Novus, NB120-6586), anti-Claudin-5 (1:400, Invitrogen, 34-1600), anti-Claudin-5 (1:200, Invitrogen 35-2500), anti-ZO-1 (1:400, Invitrogen, 61-7300), anti-AQP-4 (1:500, Millipore, AB3594), anti-PDGFRβ (1:400, Cell Signaling, 3169 S), anti-Hemoglobin (1:500, Cloud-Clone, PAB409Mu01), anti-Caveolin-1(1:400, Cell Signaling, 323AS), rabbit anti-MFSD2A (1:200, a generous gift from Dr. Chenghua Gu), and anti-Podocalyxin (1:400, R&D Systems, AF1556). Brain sections were then washed in PBS and incubated with appropriate fluorescent secondary antibodies (Invitrogen) for 1 h at room temperature. After extensive wash, the sections were mounted with DAPI Mount. All fluorescent images were captured using Nikon Eclipse Ti and/or LSM 710 confocal microscopes. Imaging processing was performed using ImageJ and Adobe Photoshop.

### TEM

Mice were anesthetized and transcardially perfused with PBS followed by 0.1 M sodium cacodylate buffer containing 4% PFA and 4% glutaraldehyde. Brain tissues were dissected out, fixed overnight, and post-fixed in 1% osmium tetroxide and 1% potassium ferrocyanide. Next, the collected brain tissues were *en bloc* stained with 2% uranyl acetate, dehydrated, and embedded in resin. An RMC MT-X microtome (Boeckeler Instruments) was used to cut ultra-thin sections, which were post-stained with 2% uranyl acetate and 1% lead citrate. Sections were examined and photographed using JEOL JEM1011 (JEOL) at 80 kV.

### Angioarchitecture analysis

The open-source software “Angiotool” (National Cancer Institute, USA) was used to assess brain angioarchitecture [28]. Briefly, brain sections were subjected to Podocalyxin staining. Thresholding techniques were applied to eliminate small particles, ensuring the quantification of actual vessels. Four parameters, including total vessel length, vessel density, branching index, and lacunarity, were calculated using the “Angiotool” software. Total vessel length is defined as the sum of Euclidean distances between pixels of all vessels; vessel density is expressed as the percentage of the area occupied by vessels within the explant area; branching index is defined as the number of vessel junctions per unit area; and lacunarity, an index for vascular structural nonuniformity, is reported as the average lacunarity over all box sizes.

### Image analyses

In this study, z-projection images were used for analyses. Analyses were performed in three different brain regions (cortex, hippocampus, and striatum). Two random images from each section, eight serial sections evenly distributed along the rostral-to-caudal axis from each brain, and five mice were used for quantifications. Data analysis was performed by investigators blinded for the genotypes.

For Laminin, Nidogen, Collagen IV, Caveolin-1, and MFSD2a, mean fluorescent intensity was calculated using ImageJ. For tight junction protein (TJP) expression, their coverage and contact were calculated using the Angiotool software. Specifically, TJP coverage was quantified as the percentage of TJP-positive fluorescent area over Podocalyxin-positive capillary area. TJP contact was quantified as the percentage of TJP-positive fluorescent length over Podocalyxin-positive capillary length. Similarly, pericyte coverage and contact were quantified as the percentages of PDGFRβ-positive fluorescent area and length over Podocalyxin-positive capillary area and length were quantified, respectively. AQP-4 coverage and contact were quantified as the percentages of AQP-4-positive fluorescent area and length over Podocalyxin-positive capillary area and length, respectively. For endothelial vesicles, the number of endothelial pinocytotic vesicles in TEM images was manually counted and normalized to endothelial area. In this analysis, twenty-five to twenty-seven capillaries from three mice were used for quantification.

### Statistical analyses

All statistical analyses were performed using the GraphPad Prism 10 software. The non-parametric Mann-Whitney U test was used to more accurately determine differences between two groups due to relatively low biological replicates. Significance was set at *p* < 0.05. Data were presented as mean ± SD.

## Results

### Laminin-α5 is ablated in endothelial and mural cells in α5-TPKO mice

**Fig. 1:**
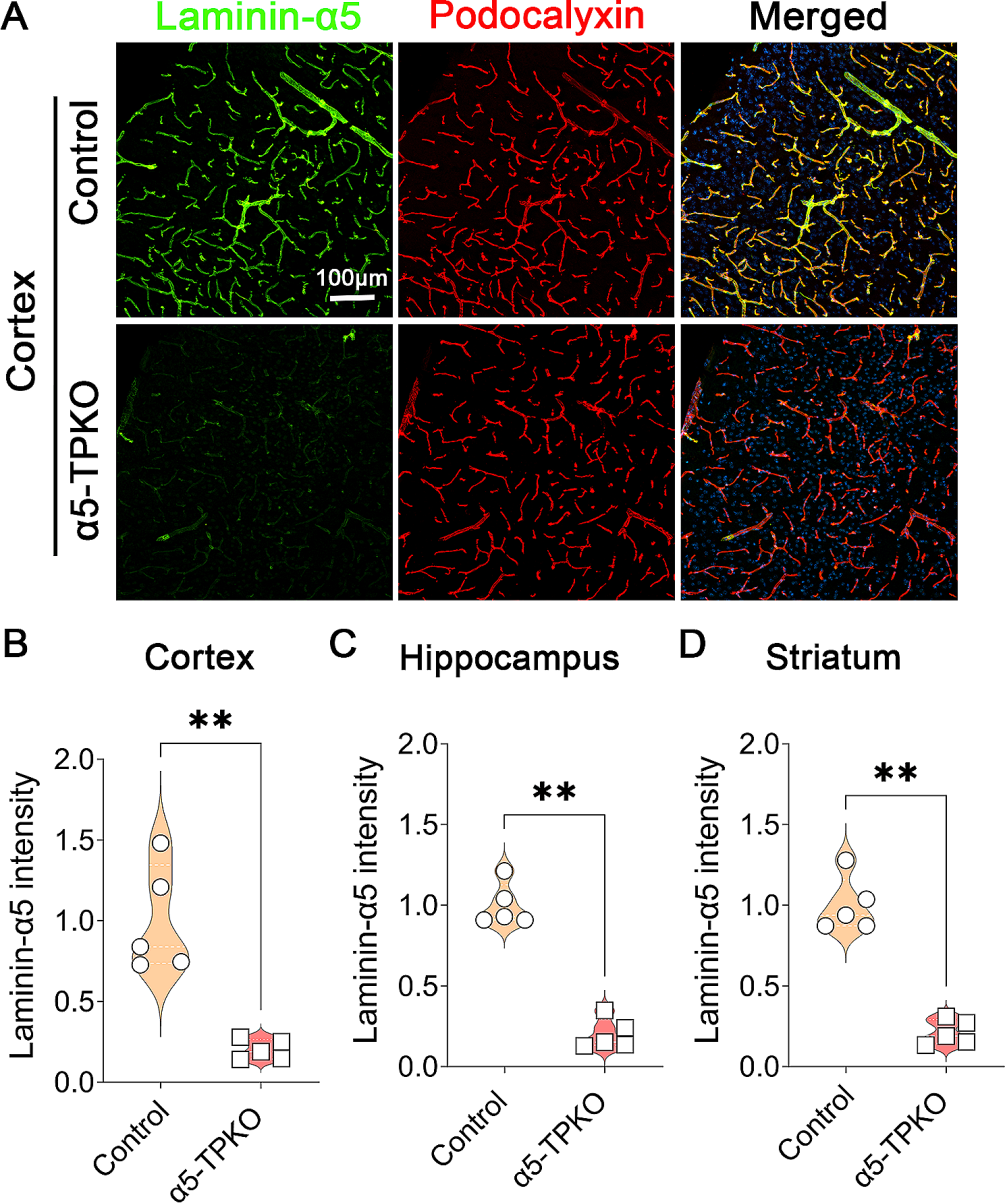
Laminin α5 expression is ablated in endothelial and mural cells in α5-TPKO mice. **A** Representative images of Laminin-α5 (green) and Podocalyxin (red) staining in the cortex of 4 ∼ 6-month-old control and α5-TPKO mice (both sexes). Scale bar: 100 μm. **B-D** Quantification of Laminin-α5 fluorescent intensity in the cortex (**B**), hippocampus (**C**), and striatum (**D**). **p = 0.0079, Mann-Whitney U test. *n* = 5 mice. Data were shown as mean ± SD

To generate mutants (α5-TPKO) with laminin-α5 deficiency in both endothelial and mural cells, we crossed laminin-α5 floxed mice with Tie2-Cre and PDGFRβ-Cre lines, which can be distinguished by using promoter-specific Cre primers. The α5-TPKO mice were born at the expected Mendelian ratio. Next, we performed immunohistochemical analysis against laminin-α5 to validate its deletion in α5-TPKO mice. Robust laminin-α5 signal, which co-localized well with vascular marker Podocalyxin, was observed in the cortex of control brains (Fig. 1A). Consistent with previous reports that endothelial and mural cells are the major cellular source of laminin-α5 in the CNS [4, 5], markedly reduced laminin-α5 level was found in the cortex of α5-TPKO brains (Fig. 1A). Quantification revealed a significant reduction in laminin-α5 expression in the cortex of α5-TPKO mice compared to that of control mice (Fig. 1B). Similar results were observed in the hippocampus (Fig. 1C) and striatum (Fig. 1D). The residual levels of laminin-α5 in α5-TPKO brains may be from other CNS cells, including neurons and oligodendrocytes, which have been reported to synthesize laminin-α5 [29–32]. These findings suggest that laminin-α5 is successfully ablated in endothelial and mural cells in α5-TPKO brains.

**Fig. 2:**
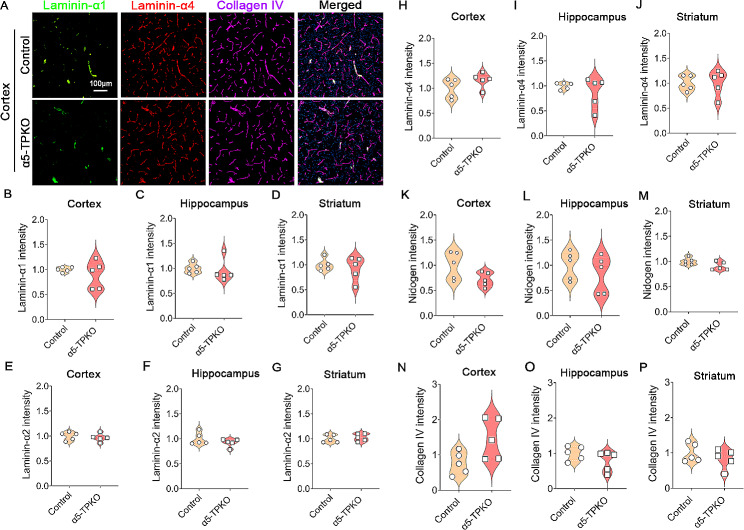
Other laminin subunits and other BL components are unaffected in α5-TPKO mice. **A** Representative images of Laminin-α1 (green), Laminin-α4 (red), and Collagen IV (magenta) staining in the cortex of 4 ∼ 6-month-old control and α5-TPKO mice (both sexes). Scale bar: 100 μm. **B-P** Quantifications of Laminin-α1 (**B-D**), Laminin-α2 (**E-G**), Laminin-α4 (**H-J**), Nidogen (**K-M**), and Collagen IV (**N-P**) fluorescent intensity in the cortex (**B, E, H, K, and N**), hippocampus (**C, F, I, L, and O**), and striatum (**D, G, J, M, and P**) of control and α5-TPKO mice. Non-significant, Mann-Whitney U test. *n* = 5 mice. Data were shown as mean ± SD

Previous studies have shown that ablation of laminin-α2 or -α4 affects other components of the basal lamina [14, 15, 33]. To determine if loss of laminin-α5 affects basal lamina composition, we further examined the expression of three other laminin subunits and two other components of the basal lamina. Interestingly, control and α5-TPKO mice exhibited comparable levels of laminin-α1 (Fig. 2A-D), laminin-α2 (Fig. 2E-G), laminin-α4 (Fig. 2H-J), nidogen (Fig. 2K-M), and collagen IV (Fig. 2N-P) in the cortex, hippocampus, and striatum. These results strongly suggest that loss of laminin-α5 does not affect the expression of other laminin subunits or other basal lamina components.

### Neurovascular integrity is disrupted in α5-TPKO mice

**Fig. 3:**
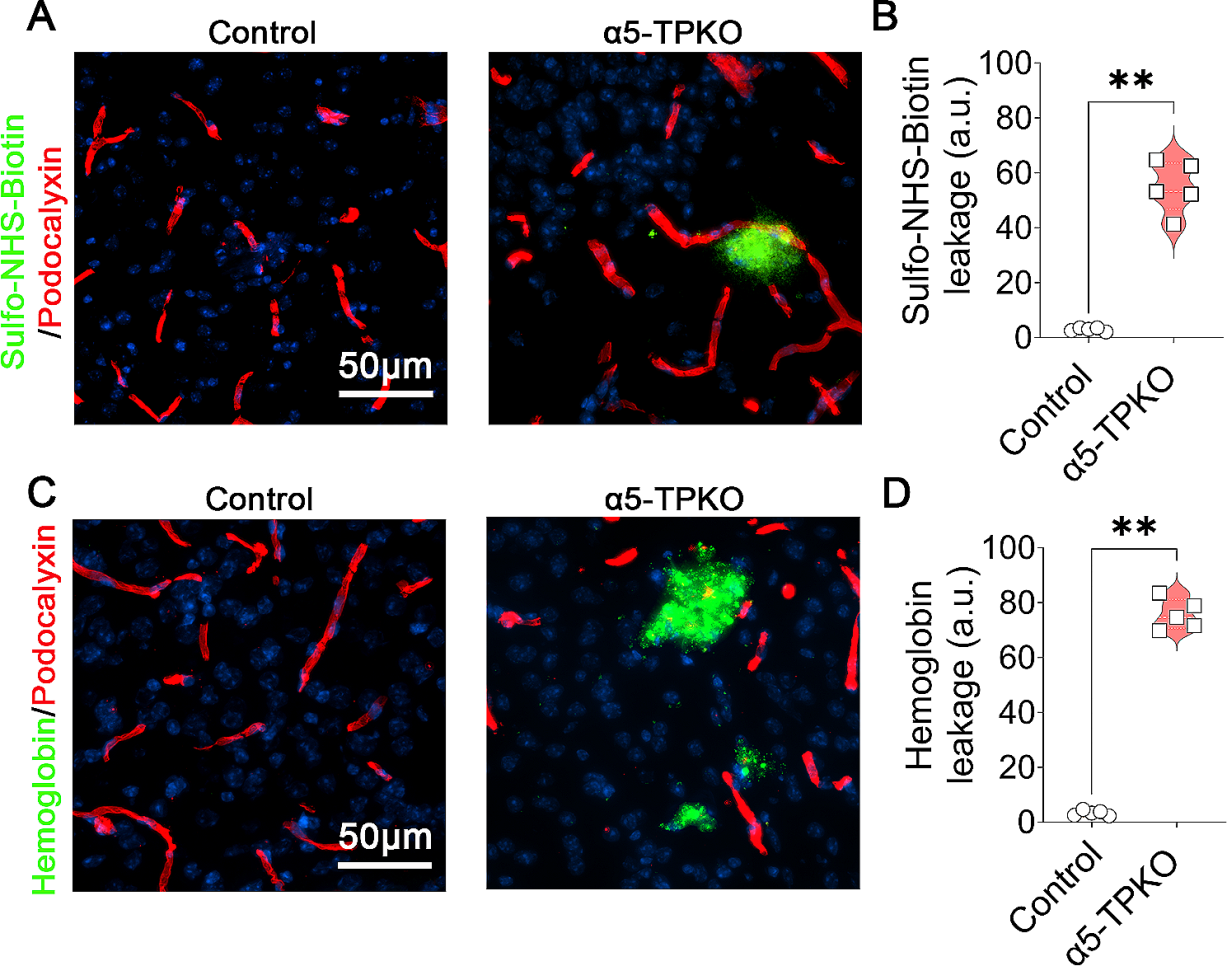
α5-TPKO mice show increased neurovascular permeability. **A** Representative images of Sulfo-NHS-Biotin (green) and Podocalyxin (red) staining in the cortex of 4 ∼ 6-month-old control and α5-TPKO mice (both sexes). Scale bar: 50 μm. **B** Quantification of Sulfo-NHS-Biotin fluorescent intensity in control and α5-TPKO brains. **p = 0.0079, Mann-Whitney U test. *n* = 5 mice. Data were shown as mean ± SD. **C** Representative images of Hemoglobin (green) and Podocalyxin (red) staining in the cortex of 4 ∼ 6-month-old control and α5-TPKO mice (both sexes). Scale bar: 50 μm. **D** Quantification of Hemoglobin fluorescent intensity in control and α5-TPKO brains. **p = 0.0079, Mann-Whitney U test. *n* = 5 mice. Data were shown as mean ± SD

**Fig. 4:**
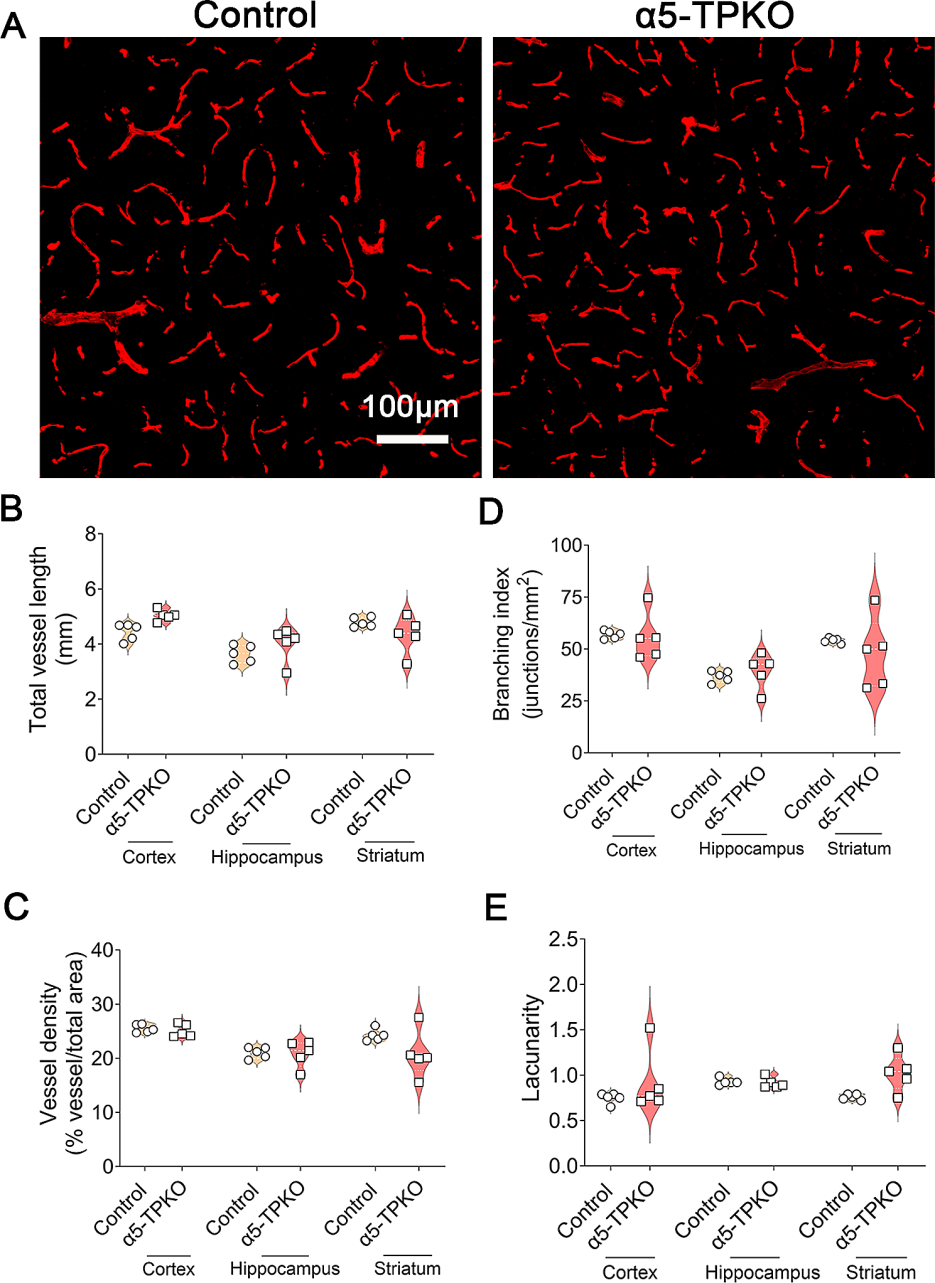
Angioarchitecture analysis for cortex, hippocampus and striatum regions. **A** Representative images of Podocalyxin staining in the cortex of 4 ∼ 6-month-old control and α5-TPKO mice (both sexes). Scale bar: 100 μm. **B-E** Quantifications of total vessel length (**B**), vessel density (**C**), branching index (**D**), and lacunarity (**E**) in the cortex, hippocampus, and striatum of control and α5-TPKO mice. Non-significant, Mann-Whitney U test. *n* = 5 mice. Data were shown as mean ± SD

To assess neurovascular integrity in α5-TPKO mice, we performed in vivo permeability assay using Sulfo-NHS-Biotin, a small exogenous tracer. Although undetectable in control brains, Sulfo-NHS-Biotin was observed in α5-TPKO brains (Fig. 3A). Quantification revealed substantially increased level of Sulfo-NHS-Biotin in α5-TPKO brains (Fig. 3B). Similarly, Hemoglobin, a large endogenous tracer, was found in α5-TPKO but not control brains (Fig. 3C and D). These results strongly indicate that neurovascular integrity is compromised in α5-TPKO mice. It should be noted that Sulfo-NHS-Biotin and Hemoglobin signals did not show a homogenous/diffuse pattern in mutant brains. Instead, tracer leakage was only detected in a few specific spots. This sporadic pattern suggests that other factors (e.g. other laminin isoforms/ECM proteins) may compensate for the loss of endothelial and mural laminin-α5 in a region-specific manner. To determine if ablation of laminin-α5 in both endothelial and mural cells affects angiogenesis and/or vascular structure, we performed angioarchitecture analysis using Podocalyxin staining (Fig. 4A). Interestingly, control and α5-TPKO mice displayed comparable total vessel length (Fig. 4B), vessel density (Fig. 4C), branching index (Fig. 4D), and lacunarity (Fig. 4E) in the cortex, hippocampus, and striatum. These findings suggest that loss of laminin-α5 in endothelial and mural cells does not affect brain angioarchitecture, and that increased neurovascular permeability in α5-TPKO mice is not caused by abnormal vascular structure.

### Endothelial paracellular mechanism is unaltered in α5-TPKO brains

**Fig. 5:**
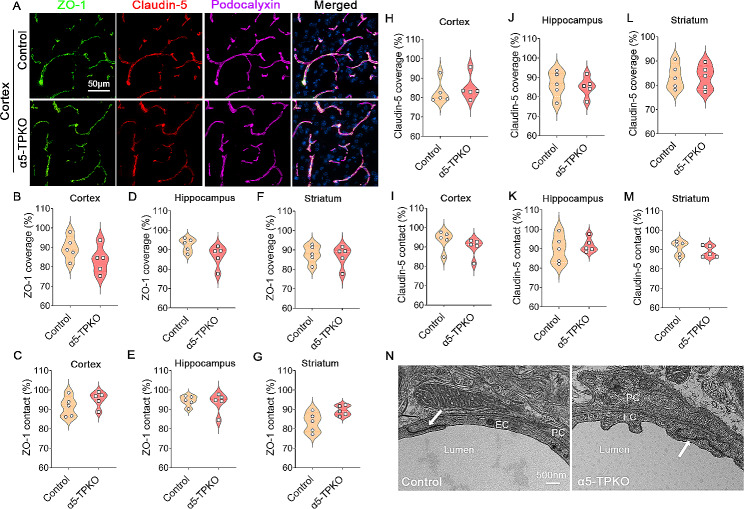
Endothelial paracellular mechanism is unaltered in α5-TPKO mice. **A** Representative images of ZO-1 (green), Claudin-5 (red), and Podocalyxin (magenta) staining in the cortex of 4 ∼ 6-month-old control and α5-TPKO mice (both sexes). Scale bar: 50 μm. **B-G** Quantifications of ZO-1 coverage (**B, D, and F**) and contact (**C, E, and G**) in the cortex (**B and C**), hippocampus (**D and E**), and striatum (**F and G**) of control and α5-TPKO mice. Non-significant, Mann-Whitney U test. *n* = 5 mice. Data were shown as mean ± SD. **H-M** Quantifications of Claudin-5 coverage (**H, J, and L**) and contact (**I, K, and M**) in the cortex (**H and I**), hippocampus (**J and K**), and striatum (**L and M**) of control and α5-TPKO mice. Non-significant, Mann-Whitney U test. *n* = 5 mice. Data were shown as mean ± SD. **N** Representative TEM images showing endothelial tight junctions in 4 ∼ 6-month-old control and α5-TPKO mice (both sexes). Arrows indicate tight junctions. PC, pericyte; EC, endothelial cell; Scale bar: 500 nm

One unique feature of brain endothelial cells is their expression of tight junction proteins, including ZO-1 and Claudin-5, which seal gaps between adjacent endothelial cells and form tight junctions [1, 34, 35]. To determine if increased neurovascular permeability in α5-TPKO mice is due to this mechanism, we first examined the expression of ZO-1 and Claudin-5 in control and α5-TPKO brains. Immunohistochemical analysis showed that ZO-1 and Claudin-5 co-localized with Podocalyxin in both control and α5-TPKO brains (Fig. 5A). Quantifications revealed comparable ZO-1 coverage and contact in the cortex (Fig. 5B and C), hippocampus (Fig. 5D and E), and striatum (Fig. 5F and G) of control and α5-TPKO mice. Similar to ZO-1, no significant difference in Claudin-5 expression was found in control and α5-TPKO mice at all three brain regions (Fig. 5H-M). These results indicate a negligible role of laminin-α5 in TJP expression. Next, we further investigated the ultrastructure of tight junctions using TEM. Electron-dense tight junctions were observed in both control and α5-TPKO brains (Fig. 5N), highlighting intact tight junctions. Together, these findings suggest that endothelial paracellular mechanism is unaffected in α5-TPKO brains.

### Endothelial transcellular permeability is increased in α5-TPKO brains

**Fig. 6:**
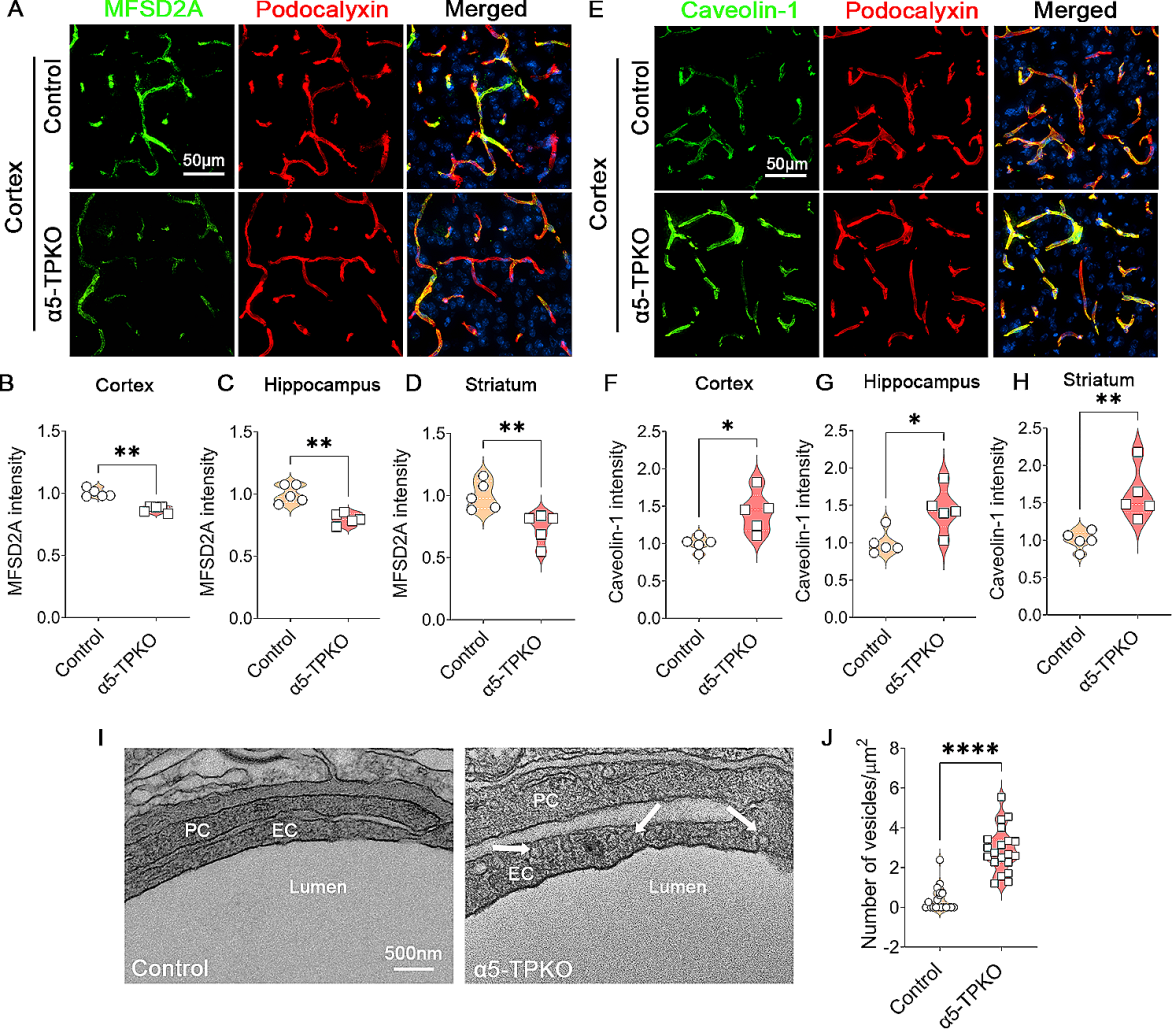
Endothelial transcellular permeability is increased in α5-TPKO mice. **A** Representative images of MFSD2a (green) and Podocalyxin (red) staining in the cortex of 4 ∼ 6-month-old control and α5-TPKO mice (both sexes). Scale bar: 50 μm. **B-D** Quantifications of MFSD2a fluorescent intensity in the cortex (**B**), hippocampus (**C**), and striatum (**D**) of control and α5-TPKO mice. **p = 0.0079, Mann-Whitney U test. *n* = 5 mice. Data were shown as mean ± SD. **E** Representative images of Caveolin-1 (green) and Podocalyxin (red) staining in the cortex of 4 ∼ 6-month-old control and α5-TPKO mice (both sexes). Scale bar: 50 μm. **F-H** Quantifications of Caveolin-1 fluorescent intensity in the cortex (**F**), hippocampus (**G**), and striatum (**H**) of control and α5-TPKO mice. *p = 0.0159 and **p = 0.0079, Mann-Whitney U test. *n* = 5 mice. Data were shown as mean ± SD. **I** Representative TEM images showing endothelial transcytosis in 4 ∼ 6-month-old control and α5-TPKO mice (both sexes). Arrows indicate caveolae vesicles. PC, pericyte; EC, endothelial cell; Scale bar: 500 nm. **J** Quantifications of endothelial caveolae vesicle number in control and α5-TPKO brains. ****p < 0.0001, Mann-Whitney U test. *n* = 20–22 capillaries from 3 mice

Another unique feature of brain endothelial cells is their extremely low rate of transcytosis [1, 34, 35], which is predominantly mediated by Caveolin-1. To investigate whether this mechanism is responsible for the exacerbated neurovascular leakage in α5-TPKO mice, we first examined the expression of MFSD2a, which negatively regulates transcytosis in endothelial cells [36]. Consistent with previous reports [36, 37], high levels of MFSD2a were observed in Podocalyxin-positive vessels in control brains (Fig. 6A). Reduced MFSD2a expression was found in α5-TPKO brains (Fig. 6A). Quantification revealed a 13%, 20%, and 26% reduction of MFSD2a fluorescent intensity in the cortex (Fig. 6B), hippocampus (Fig. 6C), and striatum (Fig. 6D) of α5-TPKO mice, respectively. Next, we examined the expression of Caveolin-1, which mediates transcytosis in brain endothelial cells [37]. Compared to the controls, increased Caveolin-1 expression was detected in the cortex of α5-TPKO mice (Fig. 6E). Quantification revealed a 41%, 43%, and 61% increase of Caveolin-1 fluorescent intensity in the cortex (Fig. 6F), hippocampus (Fig. 6G), and striatum (Fig. 6H) of α5-TPKO mice, respectively. These results highlight an important role of laminin-α5 in MFSD2a and Caveolin-1 expression. Consistent with these biochemical changes, TEM analysis revealed significantly increased number of endothelial vesicles (2.9/µm^2^) in α5-TPKO brains compared to that (0.4/µm^2^) in the controls (Fig. 6I and J). Together, these findings suggest that increased endothelial transcytosis is responsible for the neurovascular leakage in α5-TPKO mice.

### Pericyte coverage but not astrocyte polarity is diminished in α5-TPKO mice

**Fig. 7:**
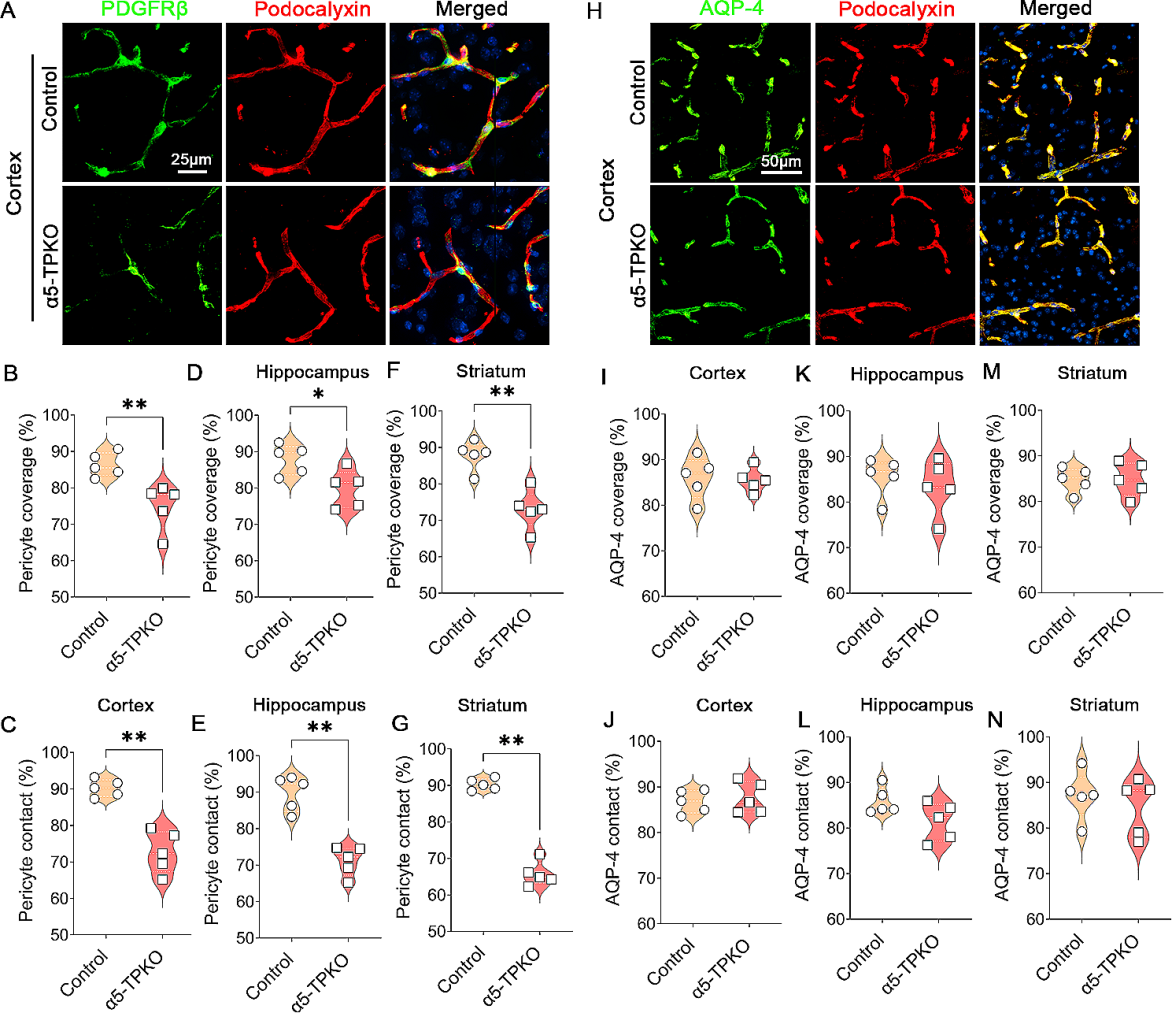
Pericyte coverage but not astrocyte polarity is diminished in α5-TPKO mice. **A** Representative images of PDGFRβ (green) and Podocalyxin (red) staining in the cortex of 4 ∼ 6-month-old control and α5-TPKO mice (both sexes). Scale bar: 25 μm. **B-G** Quantifications of pericyte coverage (**B, D, and F**) and contact (**C, E, and G**) in the cortex (**B and C**), hippocampus (**D and E**), and striatum (**F and G**) of control and α5-TPKO mice. *p = 0.0159 and **p = 0.0079, Mann-Whitney U test. *n* = 5 mice. Data were shown as mean ± SD. **H** Representative images of AQP-4 (green) and Podocalyxin (red) staining in the cortex of 4 ∼ 6-month-old control and α5-TPKO mice (both sexes). Scale bar: 50 μm. **I-N** Quantifications of AQP-4 coverage (**I, K, and M**) and contact (**J, L, and N**) in the cortex (**I and J**), hippocampus (**K and L**), and striatum (**M and N**) of control and α5-TPKO mice. non-significant, Mann-Whitney U test. *n* = 5 mice. Data were shown as mean ± SD

Pericyte coverage on capillaries plays an important role in maintaining neurovascular integrity [38, 39]. To determine if pericyte coverage is altered in α5-TPKO brains, we performed immunohistochemical analysis against PDGFRβ and Podocalyxin (Fig. 7A). Consistent with the neurovascular permeability data, quantification revealed significantly decreased pericyte coverage on capillaries in the cortex (74.9% vs. 86.3% in controls, Fig. 7B), hippocampus (79.9% vs. 87.9% in controls, Fig. 7D), and striatum (73.0% vs. 87.9% in controls, Fig. 7F) of α5-TPKO mice. Similarly, α5-TPKO mice exhibited significantly reduced pericyte contact on capillaries in the cortex (72.7% vs. 90.2% in controls, Fig. 7C), hippocampus (71.2% vs. 89.8% in controls, Fig. 7E), and striatum (65.7% vs. 90.3% in controls, Fig. 7G). These results indicate an essential role of laminin-α5 in the maintenance of pericyte coverage on capillaries.

Astrocytes wrap endothelial cells and pericytes with their endfeet. It has been shown that polarized expression of aquaporin-4 (AQP-4) in astrocytic endfeet correlates with neurovascular integrity [38, 40, 41]. To determine if astrocyte polarity is changed in α5-TPKO brains, we performed immunohistochemical analysis against AQP-4 and Podocalyxin (Fig. 7H). Interestingly, control and α5-TPKO mice exhibited comparable AQP-4 coverage (Fig. 7I, K, and M) and contact (Fig. 7J, L, and N) on capillaries in the cortex (Fig. 7I and J), hippocampus (Fig. 7K and L), and striatum (Fig. 7M and N). These results highlight a minimal role of laminin-α5 in astrocyte polarity.

## Discussion

This work provides novel and significant insights on the functional significance of laminin-α5 in neurovascular integrity. In the CNS, laminin-α5 is mainly produced by endothelial and mural cells. Previous loss-of-function studies failed to detect neurovascular leakage in mutant mice with laminin-α5 deficiency in endothelial cells [22] or mural cells [23] alone. It was speculated that loss of laminin-α5 from one cell type may be compensated by other laminin subunits (e.g. laminin-α4). However, another possibility is that loss of laminin-α5 from one cell type may be compensated by laminin-α5 from other cells. Using a compound mutant mouse line, this study clearly shows that loss of laminin-α5 from endothelial and mural cells simultaneously leads to increased neurovascular permeability, providing the first in vivo evidence that laminin-α5 actively maintains neurovascular integrity. It should be noted that we cannot exclude the possibility of potential compensation by other laminin subunits in α5-TPKO mice. However, such compensation is insufficient to maintain neurovascular integrity when laminin-α5 is ablated in both endothelial and mural cells, highlighting a possibly more important role of laminin-α5 in neurovascular integrity maintenance. These results are consistent with previous studies showing the inhibitory role of laminin-α5 in leukocyte extravasation in vivo [33, 42] and barrier function in vitro [43, 44].

Subsequent mechanistic study demonstrated that neurovascular leakage in α5-TPKO mice is mainly mediated by the transcellular rather than paracellular mechanism (Figs. 5 and 6). Like α5-TPKO mice, mutants lacking laminin-γ1 (both laminin-411 and − 511) in endothelial cells exhibit increased neurovascular permeability predominantly due to increased transcytosis. In sharp contrast, mutants with laminin-γ1 deficiency in astrocytes (laminin-211) [12, 13] and laminin-α2 global knockout mice [14] develop neurovascular leakage with tight junction abnormalities, indicating increased paracellular leakage. Interestingly, mice missing laminin-γ1 in mural cells display age-dependent neurovascular leakage, and both paracellular and transcellular mechanisms are affected in these mutants [10]. These results suggest that different laminin isoforms may regulate neurovascular integrity via distinct mechanisms. Understanding the functional significance of each laminin isoform in neurovascular integrity and their molecular mechanisms will fill the gap of knowledge in the field and open doors for new research.

Accumulating evidence supports an indispensable role of pericytes in transcellular leakage. For example, pericyte-deficient mice develop increased endothelial vesicle trafficking without obvious junction defects [38, 39, 45, 46]. It has also been reported that pericytes regulates neurovascular permeability partially via Mfsd2a, a negative regulator of caveolae-mediated transcytosis [36, 37]. Similarly, reduced pericyte coverage increases neurovascular permeability and endothelial transcytosis without affecting TJPs following chronic cerebral hypoperfusion [47]. In addition, a recent study shows that pericytes regulate endothelial transcytosis via the vitronectin-integrin-α5 signaling pathway [48]. Furthermore, loss of pericyte-derived laminin-γ1 leads to enhanced transcellular leakage and mild neurovascular leakage in an age-dependent manner [10], suggesting that pericytes may regulate neurovascular integrity via secreting laminin-γ1. Consistent with these reports, we found substantially reduced pericyte coverage in α5-TPKO mice, highlighting a critical role of laminin-α5 in pericyte coverage. It remains unclear how laminin-α5 regulates pericyte coverage and neurovascular integrity. One possibility is that laminin-α5 directly signals to pericytes and modulates their biology (e.g. vitronectin and/or laminin-γ1 expression), which affects transcytosis in endothelial cells, leading to increased neurovascular permeability. Another possibility is that laminin-α5 binds to receptors on endothelial cells to regulate their transcytosis, which affects pericyte coverage indirectly. Alternatively, laminin-α5 may act on both pericytes and endothelial cells. These important questions will be addressed in future research.

One limitation of this study is that the receptors and signaling pathways mediating laminin-α5’s effects in endothelial and mural cells remain unknown. Previous studies have shown that endothelial cells mainly express integrin-α3β1, -α6β1, -α6β4, -α5, -αv, and -β3 under physiological conditions; while mural cells predominantly synthesize integrin-α1β1, -α2β1, -α5β1, -α6β1, and -αv [4, 5]. Among these receptors, integrin-α3β1, -α6β1, and -α6β4 are classical laminin-binding integrin receptors [6, 49]. Thus, their functional significance in neurovascular integrity maintenance will be first explored in the future. Another limitation of this study lies in the Cre lines used in this study. Given that laminin is very long-lived with an extremely low turnover rate [50–53] and it takes months or years to degrade laminin synthesized before tamoxifen injection, we are unable to use tamoxifen-inducible CreER lines for laminin ablation. In this study, Tie2-Cre and PDGFRβ-Cre lines were used to ablate laminin-α5 in endothelial cells and mural cells, respectively. Although Tie2-Cre leaks into hematopoietic cells [54, 55], hematopoietic cell-derived laminin-α5 is unlikely to regulate neurovascular integrity. First, hematopoietic cells, located in the periphery, are unable to deposit laminin in the neurovascular unit under homeostatic conditions. More importantly, our unpublished data show that hematopoietic cell-derived γ1-containing laminins, which include most α5-containing laminins [6], do not contribute to neurovascular integrity maintenance. In addition to mural cells, PDGFRβ-Cre also labels fibroblasts in the CNS [56–58]. However, fibroblast-derived laminin-α5 is unlikely to maintain neurovascular integrity, since fibroblasts are found in large blood vessels rather than capillaries in the CNS [56, 57]. A third limitation of this study is that the compensatory mechanism upon loss of endothelial and mural laminin-α5 is largely known. Answer to this question may explain why tracer leakage in α5-TPKO mice occurs at specific sites rather than in all areas with diminished laminin-α5 expression.

## Conclusions

In conclusion, we show that endothelial and mural cell-derived laminin-α5 actively maintains neurovascular integrity via the transcellular rather than paracellular mechanism. These findings suggest that laminin-α5 signaling and/or transcytosis-associated molecules may be targeted to modulate neurovascular integrity.

## Data Availability

No datasets were generated or analysed during the current study.

## Abbreviations

α5-TPKO: Laminin-α5^flox/flox^-PDGFRβ-Cre^+^;Tie2-Cre^+^
AQP-4: Aquaporin-4
MFSD2A: Major facilitator superfamily domain-containing protein 2a
NVU: Neurovascular Unit
PDGFRβ: Platelet-derived growth factor receptor beta
TEM: Transmission electron microscopy
TJP: Tight junction protein
ZO-1: Zonula occluden-1

## References

[1] Langen UH, Ayloo S, Gu C (2019). Development and Cell Biology of the blood-brain barrier. Annu Rev Cell Dev Biol.

[2] Sweeney MD, Zhao Z, Montagne A, Nelson AR, Zlokovic BV (2019). Blood-brain barrier: from physiology to Disease and back. Physiol Rev.

[3] Obermeier B, Daneman R, Ransohoff RM (2013). Development, maintenance and disruption of the blood-brain barrier. Nat Med.

[4] Nirwane A, Yao Y (2022). Cell-specific expression and function of laminin at the neurovascular unit. J Cereb Blood Flow Metab.

[5] Nirwane A, Yao Y (2019). Laminins and their receptors in the CNS. Biol Rev Cam Philos Soc.

[6] Yao Y (2017). Laminin: loss-of-function studies. Cell Mol Life Sci.

[7] Sixt M, Engelhardt B, Pausch F, Hallmann R, Wendler O, Sorokin LM (2001). Endothelial cell laminin isoforms, laminins 8 and 10, play decisive roles in T cell recruitment across the blood-brain barrier in experimental autoimmune encephalomyelitis. J Cell Biol.

[8] Jucker M, Tian M, Norton DD, Sherman C, Kusiak JW (1996). Laminin alpha 2 is a component of brain capillary basement membrane: reduced expression in dystrophic dy mice. Neuroscience.

[9] Sorokin LM, Pausch F, Frieser M, Kroger S, Ohage E, Deutzmann R (1997). Developmental regulation of the laminin alpha5 chain suggests a role in epithelial and endothelial cell maturation. Dev Biol.

[10] Gautam J, Cao Y, Yao Y (2020). Pericytic laminin maintains blood-brain Barrier Integrity in an age-dependent manner. Transl Stroke Res.

[11] Biswas S, Shahriar S, Giangreco NP, Arvanitis P, Winkler M, Tatonetti NP (2022). Mural Wnt/beta-catenin signaling regulates Lama2 expression to promote neurovascular unit maturation. Development.

[12] Yao Y, Chen ZL, Norris EH, Strickland S (2014). Astrocytic laminin regulates pericyte differentiation and maintains blood brain barrier integrity. Nat Commun.

[13] Chen ZL, Yao Y, Norris EH, Kruyer A, Jno-Charles O, Akhmerov A (2013). Ablation of astrocytic laminin impairs vascular smooth muscle cell function and leads to hemorrhagic stroke. J Cell Biol.

[14] Menezes MJ, McClenahan FK, Leiton CV, Aranmolate A, Shan X, Colognato H (2014). The Extracellular matrix protein laminin alpha2 regulates the maturation and function of the blood-brain barrier. J Neurosci.

[15] Thyboll J, Kortesmaa J, Cao R, Soininen R, Wang L, Iivanainen A (2002). Deletion of the laminin alpha4 chain leads to impaired microvessel maturation. Mol Cell Biol.

[16] Miner JH, Cunningham J, Sanes JR (1998). Roles for laminin in embryogenesis: exencephaly, syndactyly, and placentopathy in mice lacking the laminin alpha5 chain. J Cell Biol.

[17] Luo S, Liu ZG, Wang J, Luo JX, Ye XG, Li X (2022). Recessive LAMA5 variants Associated with partial Epilepsy and spasms in Infancy. Front Mol Neurosci.

[18] Sampaolo S, Napolitano F, Tirozzi A, Reccia MG, Lombardi L, Farina O (2017). Identification of the first dominant mutation of LAMA5 gene causing a complex multisystem syndrome due to dysfunction of the extracellular matrix. J Med Genet.

[19] Barad M, Csukasi F, Bosakova M, Martin JH, Zhang W, Paige Taylor S (2020). Biallelic mutations in LAMA5 disrupts a skeletal noncanonical focal adhesion pathway and produces a distinct bent bone dysplasia. EBioMedicine.

[20] Maselli RA, Arredondo J, Vazquez J, Chong JX, Bamshad MJ, Nickerson DA (2018). A presynaptic congenital myasthenic syndrome attributed to a homozygous sequence variant in LAMA5. Ann N Y Acad Sci.

[21] Jones LK, Lam R, McKee KK, Aleksandrova M, Dowling J, Alexander SI (2020). A mutation affecting laminin alpha 5 polymerisation gives rise to a syndromic developmental disorder. Development.

[22] Gautam J, Miner JH, Yao Y (2019). Loss of endothelial laminin alpha5 exacerbates hemorrhagic brain Injury. Transl Stroke Res.

[23] Nirwane A, Johnson J, Nguyen B, Miner JH, Yao Y (2019). Mural cell-derived laminin-alpha5 plays a detrimental role in ischemic stroke. Acta Neuropathol Commun.

[24] Nguyen NM, Kelley DG, Schlueter JA, Meyer MJ, Senior RM, Miner JH (2005). Epithelial laminin alpha5 is necessary for distal epithelial cell maturation, VEGF production, and alveolization in the developing murine lung. Dev Biol.

[25] Cuttler AS, LeClair RJ, Stohn JP, Wang Q, Sorenson CM, Liaw L (2011). Characterization of Pdgfrb-cre transgenic mice reveals reduction of ROSA26 reporter activity in remodeling arteries. Genesis.

[26] Xu L, Nirwane A, Xu T, Kang M, Devasani K, Yao Y (2022). Fibroblasts repair blood-brain barrier damage and hemorrhagic brain injury via TIMP2. Cell Rep.

[27] Gautam J, Xu L, Nirwane A, Nguyen B, Yao Y (2020). Loss of mural cell-derived laminin aggravates hemorrhagic brain injury. J Neuroinflammation.

[28] Zudaire E, Gambardella L, Kurcz C, Vermeren S (2011). A computational tool for quantitative analysis of vascular networks. PLoS ONE.

[29] Zhang Y, Chen K, Sloan SA, Bennett ML, Scholze AR, O’Keeffe S (2014). An RNA-sequencing transcriptome and splicing database of glia, neurons, and vascular cells of the cerebral cortex. J Neurosci.

[30] Kim WK, Kim D, Cui J, Jang HH, Kim KS, Lee HJ (2014). Secretome analysis of human oligodendrocytes derived from neural stem cells. PLoS ONE.

[31] Omar MH, Campbell MK, Xiao X, Zhong Q, Brunken WJ, Miner JH (2017). CNS neurons Deposit laminin alpha5 to stabilize synapses. Cell Rep.

[32] Kang M, Nirwane A, Ruan J, Adithan A, Gray M, Xu L et al. A dispensable role of oligodendrocyte-derived laminin-alpha5 in brain homeostasis and intracerebral hemorrhage. J Cereb Blood Flow Metab. 2024; In press. 10.1177/0271678X241228058. 10.1177/0271678X241228058 PMC10981398 38241459

[33] Wu C, Ivars F, Anderson P, Hallmann R, Vestweber D, Nilsson P (2009). Endothelial basement membrane laminin α5 selectively inhibits T lymphocyte extravasation into the brain. Nat Med.

[34] Chow BW, Gu C (2015). The molecular constituents of the blood-brain barrier. Trends Neurosci.

[35] Knowland D, Arac A, Sekiguchi KJ, Hsu M, Lutz SE, Perrino J (2014). Stepwise recruitment of transcellular and paracellular pathways underlies blood-brain barrier breakdown in stroke. Neuron.

[36] Ben-Zvi A, Lacoste B, Kur E, Andreone BJ, Mayshar Y, Yan H (2014). Mfsd2a is critical for the formation and function of the blood-brain barrier. Nature.

[37] Andreone BJ, Chow BW, Tata A, Lacoste B, Ben-Zvi A, Bullock K (2017). Blood-brain barrier permeability is regulated by lipid transport-dependent suppression of Caveolae-Mediated Transcytosis. Neuron.

[38] Armulik A, Genove G, Mae M, Nisancioglu MH, Wallgard E, Niaudet C (2010). Pericytes regulate the blood-brain barrier. Nature.

[39] Daneman R, Zhou L, Kebede AA, Barres BA (2010). Pericytes are required for blood-brain barrier integrity during embryogenesis. Nature.

[40] Nico B, Frigeri A, Nicchia GP, Quondamatteo F, Herken R, Errede M (2001). Role of aquaporin-4 water channel in the development and integrity of the blood-brain barrier. J Cell Sci.

[41] Nico B, Paola Nicchia G, Frigeri A, Corsi P, Mangieri D, Ribatti D (2004). Altered blood-brain barrier development in dystrophic MDX mice. Neuroscience.

[42] Song J, Zhang X, Buscher K, Wang Y, Wang H, Di Russo J (2017). Endothelial basement membrane laminin 511 contributes to endothelial junctional tightness and thereby inhibits leukocyte transmigration. Cell Rep.

[43] Kangwantas K, Pinteaux E, Penny J (2016). The extracellular matrix protein laminin-10 promotes blood-brain barrier repair after hypoxia and inflammation in vitro. J Neuroinflammation.

[44] Motallebnejad P, Azarin SM (2020). Chemically defined human vascular laminins for biologically relevant culture of hiPSC-derived brain microvascular endothelial cells. Fluids Barriers CNS.

[45] Bell RD, Winkler EA, Sagare AP, Singh I, LaRue B, Deane R (2010). Pericytes control key neurovascular functions and neuronal phenotype in the adult brain and during brain aging. Neuron.

[46] Villasenor R, Kuennecke B, Ozmen L, Ammann M, Kugler C, Gruninger F (2017). Region-specific permeability of the blood-brain barrier upon pericyte loss. J Cereb Blood Flow Metab.

[47] Sun Z, Gao C, Gao D, Sun R, Li W, Wang F (2021). Reduction in pericyte coverage leads to blood-brain barrier dysfunction via endothelial transcytosis following chronic cerebral hypoperfusion. Fluids Barriers CNS.

[48] Ayloo S, Lazo CG, Sun S, Zhang W, Cui B, Gu C (2022). Pericyte-to-endothelial cell signaling via vitronectin-integrin regulates blood-CNS barrier. Neuron.

[49] Aumailley M (2013). The laminin family. Cell Adh Migr.

[50] Dankovich TM, Rizzoli SO (2022). The synaptic Extracellular Matrix: Long-Lived, stable, and still remarkably dynamic. Front Synaptic Neurosci.

[51] Beavan LA, Davies M, Couchman JR, Williams MA, Mason RM (1989). In vivo turnover of the basement membrane and other heparan sulfate proteoglycans of rat glomerulus. Arch Biochem Biophys.

[52] Cohen MP, Surma M (1980). Renal glomerular basement membrane. In vivo biosynthesis and turnover in normal rats. J Biol Chem.

[53] Trier JS, Allan CH, Abrahamson DR, Hagen SJ (1990). Epithelial basement membrane of mouse jejunum. Evidence for laminin turnover along the entire crypt-villus axis. J Clin Investig.

[54] Tang Y, Harrington A, Yang X, Friesel RE, Liaw L (2010). The contribution of the Tie2 + lineage to primitive and definitive hematopoietic cells. Genesis.

[55] Joseph C, Quach JM, Walkley CR, Lane SW, Lo Celso C, Purton LE (2013). Deciphering hematopoietic stem cells in their niches: a critical appraisal of genetic models, lineage tracing, and imaging strategies. Cell Stem Cell.

[56] Vanlandewijck M, He L, Mae MA, Andrae J, Ando K, Del Gaudio F (2018). A molecular atlas of cell types and zonation in the brain vasculature. Nature.

[57] Pietila R, Del Gaudio F, He L, Vazquez-Liebanas E, Vanlandewijck M, Muhl L (2023). Molecular anatomy of adult mouse leptomeninges. Neuron.

[58] Nirwane A, Yao Y (2022). SMA(low/undetectable) pericytes differentiate into microglia- and macrophage-like cells in ischemic brain. Cell Mol Life Sci.

